# Supplementary material for: Evaluation of 73 Enlisted Patients for Liver Transplant with Unknown Etiology Reveals a Late-Diagnosed Case of Lysosomal Acid Lipase Deficiency
Source: Int J Mol Sci. 2024 Aug 8;25(16):8648. doi: 10.3390/ijms25168648 (PMC11354363; doi:10.3390/ijms25168648)
Supplement: Supplementary file 1 [file ijms-25-08648-s001.zip › ijms-3115452-supplementary.pdf]

Supplementary figure S1 - Schematic representation of the *LIPA* gene highlighting the position of the likely pathogenic homozygous missense variant identified in the patient by Sanger sequencing.

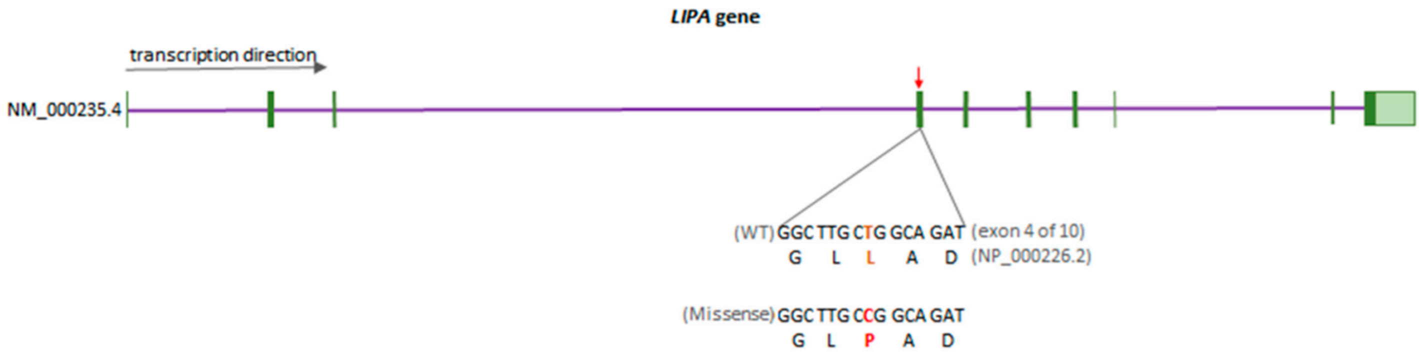

The intronic regions of *LIPA* are depicted with purple lines. Exons are represented by green boxes, with the dark green portions corresponding to the coding sequence. The position of the NM_000235.4:c.266T>C variant in exon 4 of 10 is indicated by a red arrow. This representation also schematizes the effect of this point variant on protein translation, involving a substitution of leucine (L) with proline (P) at amino acid position 89 of 400 (NP_000226.2).

Supplementary document S1 - Criteria and arguments for the *LIPA*(NM_000235).4:c.266T>C(p.Leu89Pro) classification as likely pathogenic, according to ACMG guidelines[1,2].

- PM1, because the UniProt[3] protein LICH_HUMAN (P38571) domain 'AB hydrolase-1' (80-380aa) has 175 missense/in-frame variants (49 pathogenic variants, 122 uncertain variants and four benign variants);
- PM2, because the variant is absent in gnomAD[4] and ABraOM[5] (population genomic databases);
- PM3, because it was detected in homozygous state;
- PP2, because it is a missense variant in a *LIPA* gene, which missense mutation is a common mechanism of a disease[6];
- PP3, because multiple computational prediction tools – Revel[7], MetaRNN[8], MutPred[9], EIGEN[10], FATHMM-XF[11], and SIFT[12] - support a deleterious effect for this variant;
- PP5, because ClinVar[13] classifies this variant as likely pathogenic, with one star (reviewed January 2024; one submission).

References

1. Richards S, Aziz N, Bale S, Bick D, Das S, Gastier-Foster J, et al. Standards and guidelines for the interpretation of sequence variants: A joint consensus recommendation of the American College of Medical Genetics and Genomics and the Association for Molecular Pathology. Genet Med [internet]. 2015;17(5):405–24;

2. Tavtigian S V., Harrison SM, Boucher KM, Biesecker LG. Fitting a naturally scaled point system to the ACMG/AMP variant classification guidelines. Hum Mutat. 2020;41(10):1734–7; https://doi.org/10.1002/humu.24088

3. Bateman A, Martin M-J, Orchard S, Magrane M, Ahmad S, Alpi E, et al. UniProt: the Universal Protein Knowledgebase in 2023. Nucleic Acids Res. 2023;51(D1):D523–31; https://doi.org/10.1093/nar/gkac1052

4. Chen S, Francioli LC, Goodrich JK, Collins RL, Kanai M, Wang Q, et al. A genomic mutational constraint map using variation in 76,156 human genomes. Nature. 2024;625(7993):92–100; https://doi.org/10.1038/s41586-023-06045-0

5. Naslavsky MS, Scliar MO, Yamamoto GL, Wang JYT, Zverinova S, Karp T, et al. Whole-genome sequencing of 1,171 elderly admixed individuals from São Paulo, Brazil. Nat Commun. 2022;13(1):1004; https://doi.org/10.1038/s41467-022-28648-3

6. Vinje T, Laerdahl JK, Bjune K, Leren TP, Strøm TB. Characterization of the mechanisms by which missense mutations in the lysosomal acid lipase gene disrupt enzymatic activity. Hum Mol Genet. 2019;28(18):3043–52; https://doi.org/10.1093/hmg/ddz114

7. Ioannidis NM, Rothstein JH, Pejaver V, Middha S, McDonnell SK, Baheti S, et al. REVEL: An Ensemble Method for Predicting the Pathogenicity of Rare Missense Variants. Am J Hum Genet. 2016;99(4):877–85; https://doi.org/10.1016/j.ajhg.2016.08.016

8. Li C, Zhi D, Wang K, Liu X. MetaRNN: differentiating rare pathogenic and rare benign missense SNVs and InDels using deep learning. Genome Med. 2022;14(1):115; https://doi.org/10.1186/s13073-022-01120-z

9. Pejaver V, Urresti J, Lugo-Martinez J, Pagel KA, Lin GN, Nam H-J, et al. Inferring the molecular and phenotypic impact of amino acid variants with MutPred2. Nat Commun. 2020;11(1):5918; https://doi.org/10.1038/s41467-020-19669-x

10. Ionita-Laza I, McCallum K, Xu B, Buxbaum JD. A spectral approach integrating functional genomic annotations for coding and noncoding variants. Nat Genet. 2016;48(2):214–20; https://doi.org/10.1038/ng.3477

11. Rogers MF, Shihab HA, Mort M, Cooper DN, Gaunt TR, Campbell C. FATHMM-XF: accurate prediction of pathogenic point mutations via extended features. Bioinformatics. 2018;34(3):511–3; https://doi.org/10.1093/bioinformatics/btx536

12. Vaser R, Adusumalli S, Leng SN, Sikic M, Ng PC. SIFT missense predictions for genomes. Nat Protoc. 2016;11(1):1–9; https://doi.org/10.1038/nprot.2015.123

13. Landrum MJ, Lee JM, Benson M, Brown G, Chao C, Chitipiralla S, et al. ClinVar: public archive of interpretations of clinically relevant variants. Nucleic Acids Res. 2016;44(D1):D862–8; https://doi.org/10.1093/nar/gkv1222
